# To the Editor, “Prevalence of Adverse Childhood Experiences in Children With Cystic Fibrosis at a Single Center”

**DOI:** 10.1002/ppul.71593

**Published:** 2026-03-30

**Authors:** Wadsworth A. Williams, Brittany Bedford, Susanna A. McColley

**Affiliations:** ^1^ Department of Pediatrics Ann and Robert H. Lurie Children's Hospital of Chicago Chicago Illinois USA; ^2^ Division of Pulmonary and Sleep Medicine, Department of Pediatrics Northwestern University Feinberg School of Medicine Chicago Illinois USA; ^3^ Stanley Manne Children's Research Institute Ann and Robert H. Lurie Children's Hospital of Chicago Chicago Illinois USA

## 1 Introduction

Adverse childhood experiences (ACEs) are a collection of childhood maltreatments, spanning the domains of abuse, neglect, and household dysfunction, that cause significant stress and have long‐term effects on physical and mental health [1]. ACEs lead to toxic levels of stress, which increase cortisol levels and distort the hypothalamic‐pituitary axis, resulting in long‐term health consequences that include increased risk for heart disease, diabetes, cancer, psychiatric concerns, and myriad other health conditions [2]. ACEs are common: according to recent data from National Survey of Children's Health, at least 31% of children nationally have had at least one ACE [3]. In an international meta‐analysis including more than 490,000 children from 65 studies in 18 countries, 58% of children had experienced one ACE [4]. ACEs are more prevalent in children with health needs, low socioeconomic status, and in indigenous populations [4]. There is currently no consensus on how and when to screen for ACEs, or how to intervene on positive ACEs screening [1]. Due to the lack of consensus for screening protocols and effective interventions after a positive screen, some argue that screening for ACEs should not yet occur in clinical practice [1].

There are no data documenting the prevalence of ACEs in children and young adults with cystic fibrosis (CF). Depression and anxiety, commonly associated with ACEs, are prevalent in people with CF and are associated with worse health outcomes [5, 6]. Mental health screenings are recommended in pediatric practice, and for the CF population, but screening for ACEs has lagged [1].

We previously surveyed parents at our CF center to learn about preferences for ACEs screening [7]. We found that parents at our center preferred to disclose a categorical number of, rather than specific, ACEs and that they were generally willing to participate in research and receive education about ACEs. Our aim was to conduct a pilot study to capture categorical prevalences of ACEs at a single CF center.

## 2 Methods

Parents/guardians of children and young adults with CF followed at the Ann and Robert H. Lurie Children's Hospital of Chicago CF Center were invited between February 1, 2021 and January 31, 2022 to fill out an anonymous survey (Supporting Figure 1) about their child's ACEs. Participants were offered the opportunity to participate through a survey link in their email or at clinic using a provided tablet computer. Parents/guardians of multiple children were asked to fill out the survey about their oldest child with CF. Parents filled out the survey for their oldest child, consistent with our previous study that was designed to better assess cumulative ACEs, minimize study burden, and reduce the overreporting of family‐level ACEs, for example, parental divorce [5]. Parents also preferred filling out surveys rather than having children report ACEs at any age in that study. Surveys were provided in Spanish and English and distributed in the language of preference on file with our CF center. Study data were collected and managed using REDCap electronic data capture tools hosted at Northwestern University [8]. The survey included the ACEs questionnaire constructed and validated by the Center for Youth Wellness [9]. Section 1 represents the traditional ACEs screen that is standardized in many national surveys [3]. Section 2 represents additional early life stressors that have been recognized by experts and community stakeholders [9]. These additional early life stressors are hypothesized to also disrupt the neuroendocrine axis but have not yet been correlated with large population level data [9]. The early life stressors section includes additional questions for children over the age of 13 [9]. Supporting Figure 1 displays the survey questions and response options.

We compared ACEs data from our center with both Illinois and national data from the 2021 National Survey of Children's Health. Pearson's χ [2] tests were used to compare categorical variables between participants and data from the National Survey of Children's Health. To preserve interpretability given small subgroup counts, race and ethnicity comparisons were limited. Significance was set at *p* < 0.05. Lurie Children's Institutional Review Board determined this study exempt because it was an anonymous survey of adults.

## 3 Results

The survey was completed by 66 parents, one grandparent, and one legal guardian, representing 43% of the 158 families (198 unique children and young adults with CF) with emails on file at our center during the study period. Most respondents were between 35 and 44 years of age (51.5%, 35/68), had a college or graduate degree (72.1%, 49/68), and were employed, part or full time (64.7%, 44/68). The self‐reported race and ethnicity of the respondents was generally representative of our CF Center population and of people with CF in the US: 47 (69.1%) White, 8 (11.8%) Hispanic, 6 (8.8%) Black, 4 (5.9%) White/Hispanic, and 3 (4.4%) Asian. The ages of the children the parents/guardians answered about were also representative of our center and included 17 0–6‐year‐olds, 24 7–12‐year‐olds, 21 13–18‐year‐olds, and six people with CF over 18 years of age. We found that 17.7% of children with CF had at least 1 ACE, and 17.7% had two or more ACEs (Table 1). The range of ACE exposure in our population was 0–5, with two children having three ACEs, one child with four ACEs, and one child with five ACEs. Furthermore, 46.3% of children ages 0%–12% and 59.3% of teenagers aged 13+ had at least one additional early life stressor. There were no differences in ACEs based on parental education (*p* = 0.26) or parental employment (*p* = 0.63). As displayed in Figure 1, there was no difference in the ACEs at our center and national (*p* = 0.90) or Illinois data (*p* = 0.47).

**TABLE 1:** Prevalence of ACEs and additional early life stressors at our CF center.

# of ACES	Traditional ACEs # (%)	Additional early life stressors ages 0–12 # (%)	Additional early life stressors ages 13+ # (%)
0	44 (64.7)	22 (53.7)	10 (37.0)
1	12 (17.7)	15 (36.6)	11 (40.7)
2 +	12 (17.7)	4 (9.8)	5 (18.5)
Did not respond	0 (0.0)	0 (0.0)	1 (3.7)
Total	68	41	27

**FIGURE 1:**
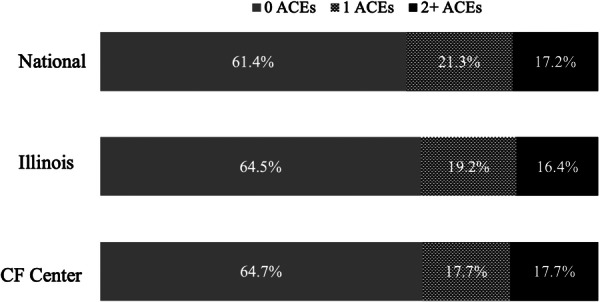
National and Illinois data are from 2021 to 2022 National Survey of Children's Health assessment of adverse childhood experiences [3].

## 4 Discussion

ACEs are as common in our CF population as state and national data during the same period. These data support that screening can be performed as part of CF Center care when conducted via electronic survey, and that ACEs are common in children and youth with CF. In addition, nearly half of children under age 12 had at least one additional early life stressor, indicating the importance of early and routine screening in this population, for a possible intervention. The 43% survey response rate was similar to that of the National Survey of Children's Health [3], though slightly below the 53% reported in a study of implementation of ACEs screening in primary care practice [10], and supports the feasibility of screening in CF clinics.

Our study adds to previous data demonstrating the prevalence of ACEs and is the first study to document prevalence of ACEs in children with CF [4]. There is, however, still debate over how and when to screen for ACEs, and how to provide resources when a positive ACEs screen occurs [1]. Although there is no consensus on how to respond to ACEs, the mainstay of interventions for ACEs is providing access to resources for families [2]. These could include assistance from a social worker, counseling through a psychologist, or referring families for resiliency training. Although these interventions may not be available in primary care settings, the US CF care center represent an ideal environment for ACEs screening and intervention since centers see children with CF frequently and have robust multidisciplinary teams that include social workers and psychologists who can intervene on positive screenings. Finally, even when a specific ACE is not disclosed, recognizing ACEs can inform discussions about unmet needs. In our previous work, we documented parental preference to learn more about, and report, ACEs. Greater awareness about ACEs can help encourage families to seek further resources to prevent and ameliorate the impacts of many of the stressors that can lead to ACEs given association of increased ACEs and special health care needs among children [4, 11]. However, there can be harms to ACEs screening if not undertaken with the appropriate sensitivity; therefore, careful consideration and planning should be undertaken at each center [12].

Limitations of this study include the single‐center study design and a relatively small sample size, reducing generalizability. There are a variety of methods to screen for ACEs – including who, when, and how the screening should be administered. Due to previously documented parental preferences, we utilized anonymous surveys, but there may be different preferred screening methods for different populations. In addition, the survey was not completed by all households and there may be selection bias in those that opted to complete the survey or those who declined to participate. There is literature to suggest that parents underestimate ACEs for their children, particularly in cases of adolescents and abuse [7]. Due to the lack of standardized protocols for screening, we previously surveyed our parental population about how they preferred to have screening, then conducted this study by delivering an anonymous, categorical screening to adult caregivers. Finally, this research study did not explore how willing parents or older children with CF are to have ACEs disclosed to CF Center professionals. Nevertheless, these data suggest that CF centers should consider screening for ACEs, seeking family input and using existing resources to moderate effects of ACEs in children with CF.

## 5 Conclusions

We found that children with CF at a single CF Center had a prevalence of ACEs similar to the general population of children in the same state and nationally. Because ACEs are common in children with CF, life‐threatening illness and serious medical procedures are early life stressors, and there are documented interventions in children with ACEs to improve health outcomes [2], ACEs screening should be considered as part of psychosocial assessment in CF Centers. However, more study is needed to further understand the best methods for screening and intervening on ACEs in CF and pediatric chronic disease populations.

## Author Contributions

**Wadsworth A. Williams II:** Conceptualization; Methodology; Data curation; Investigation; Formal analysis; Writing – original draft; Writing – review and editing; Software; Validation. **Susana A. McColley:** conceptualization, methodology, reviewing, and editing.

## Conflicts of Interest

The authors declare no conflicts of interest.

## Supporting information

Supplement_ACEs_Survey.

## Data Availability

Data available on request due to privacy/ethical restrictions.
