# Supplementary material for: To the Editor, “Prevalence of Adverse Childhood Experiences in Children With Cystic Fibrosis at a Single Center”
Source: Pediatr Pulmonol. 2026 Mar 30;61(4):e71593. doi: 10.1002/ppul.71593 (PMC13034398; doi:10.1002/ppul.71593)
Supplement: Supplementary file 1 — Supplement_ACEs_Survey. [file PPUL-61-0-s001.pdf]

Adverse Childhood Experiences Screen

A A A

Many people experience stressful life events that can affect their health and wellbeing. We would like to learn more about how these events, called "adverse childhood experiences" or ACEs, impact children with cystic fibrosis (CF). This is important because there are ways that health care teams can help children and families to reduce any negative effects that may occur. This is a screen to see how prevalent these ACEs are in our CF population.

This survey is anonymous - your name and identifying information will not be associated with your answer choices. To honor your time, we would like to give you a $10 Amazon gift certificate if you complete the survey. After completing the survey you will be directed to a separate survey to collect information for sending the gift certificate. This information will not be stored with your responses.

If you have more than one child with CF, please answer about your oldest child.

Your CF team is available in person or by phone should you wish to discuss any discomfort you feel after seeing the questions, whether or not you complete the survey.

For more information about adverse childhood experiences and why we are studying them:

Attachment: Adverse_Childhood_Experience_Info.pdf (329.3 kB)

What is your relationship to a child with CF?

- Parent
- Grandparent
- Other legal guardian
- Prefer not to answer

What is your age?

- 18-24
- 25-34
- 35-44
- 45-54

	◯ 55+<br>◯ Prefer not to answer
**What is your child's race/ethnicity?**	☐ Asian<br>☐ Black/African American<br>☐ Caucasian<br>☐ Hispanic/Latinx<br>☐ Native American<br>☐ Pacific Islander<br>☐ Other (write-in answer)<br>☐ Prefer not to answer
**What is the highest level of schooling or degree that you have *completed?***	◯ Less than a high school diploma<br>◯ High school degree or equivalent<br>◯ College degree<br>◯ Graduate degree<br>◯ Prefer not to answer
**What is your current employment status?**	◯ Employed full time (40 or more hours per week)<br>◯ Employed part time (up to 39 hours per week)<br>◯ Unemployed<br>◯ Student<br>◯ Retired<br>◯ Homemaker full time<br>◯ Unable to work<br>◯ Other (write-in answer)<br>◯ Prefer no to answer

How old is your child with CF? (If you have more than one child please choose the age of the oldest)

* must provide value

- ○ 0-6 years
- ○ 7-12 years
- ◉ 13-18 years
- ○ 18+ years
- ○ Prefer not to answer

Please read the section below and then select how many apply to your child.

Section 1. At any point since your child was born...

•Your child's parents or guardians were separated or divorced
•Your child lived with a household member who served time in jail or prison
•Your child lived with a household member who was depressed, mentally ill, or attempted suicide
•Your child saw or heard household members hurt or threaten to hurt each other
•A household member swore at, insulted, humiliated, or put down your child in a way that scared your child OR a household member acted in a way that made your child afraid that s/he might be physically hurt
•Someone touched your child's private parts or asked your child to touch their private parts in a sexual way
•More than once, your child went without food, clothing, a place to live, or had no one to protect her/him
•Someone pushed, grabbed, slapped or threw something at your child OR your child was hit so hard that your child was injured or had marks
•Your child lived with someone who had a problem with drinking or using drugs
•Your child often felt unsupported, unloved and/or unprotected

Number of ACEs from Section 1

- ○ 0
- ○ 1
- ○ 2
- ○ 3
- ○ 4
- ○ 5
- ○ 6
- ○ 7

- 8
- 9
- 10

Please read the section below and then select how many apply to your child.

<u>Section 2</u>: At any point since your child was born...

•Your child was in foster care
•Your child experienced harassment or bullying at school
•Your child lived with a parent or guardian who died
•Your child was separated from her/his primary caregiver through deportation or immigration
•Your child had a serious medical procedure or life-threatening illness
•Your child often saw or heard violence in the neighborhood or in her/his school neighborhood
•Your child was often treated badly because of race, sexual orientation, place of birth, disability or religion
•Your child was detained, arrested or incarcerated
•Your child experienced verbal or physical abuse or threats from a romantic partner (i.e. boyfriend or girlfriend)

Number of ACEs from Section 2

- 0
- 1
- 2
- 3
- 4
- 5
- 6
- 7
- 8
- 9

If you would like to be contacted by a Lurie Children's CF Center care team

member about this project or have concerns for your child, please call the CF Center at 312-227-6260 or send a note through MyChart.

<< Previous Page

Submit

Save & Return Later

Powered by REDCap

We need YOUR help understanding the impact of

Adverse Childhood Experiences (ACEs) on children with CF

What are ACEs?

Adverse childhood experiences, or "ACEs," are stressful life events that occur in childhood and can have long-term health consequences. ACEs are ***common***. The National Survey for Children's Health estimates that almost half of American children have experienced at least 1 ACE. ACEs include abuse, neglect, and stress from the household.

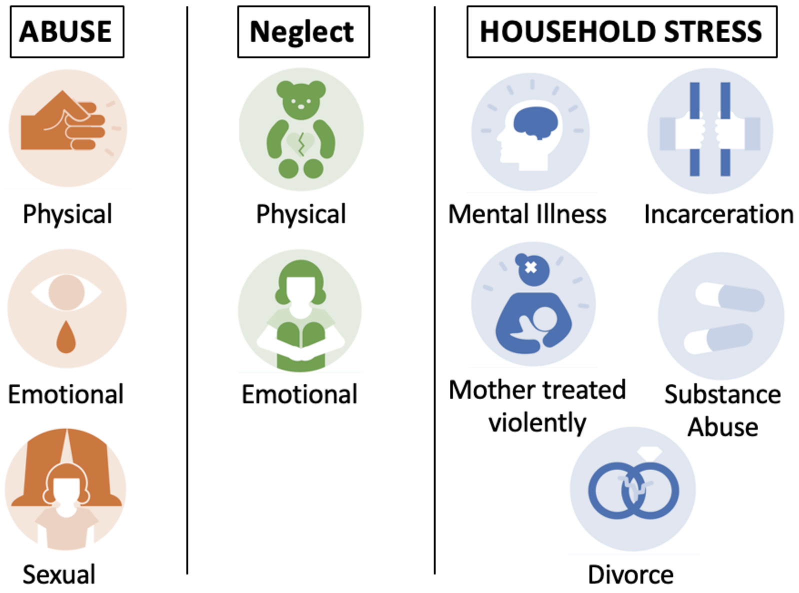

Center for Youth Wellness ACE-Q User Guide

Why do ACEs Matter?

More ACEs may lead to poorer health during childhood and adult life. ACEs in children may lead to learning and behavior problems, obesity, depressed mood, substance use, and suicide.

In 1998 the Centers for Disease Control (CDC) studied thousands of adults and found those that who reported 4+ ACEs had much worse health.

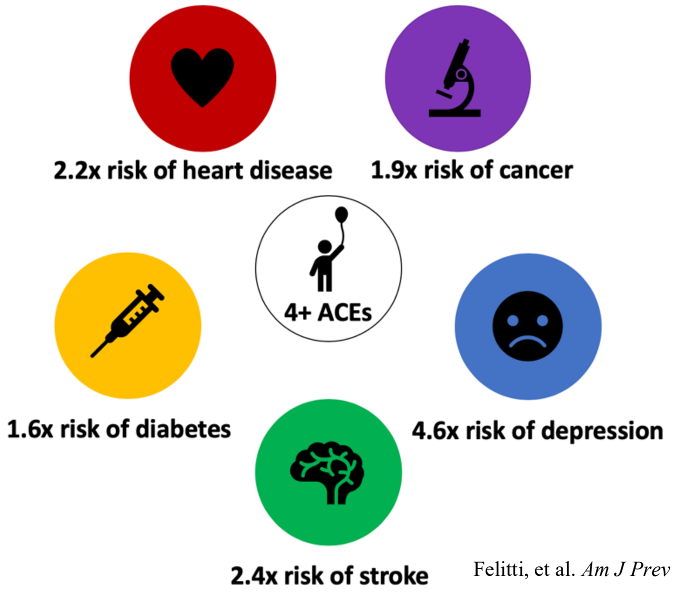

Felitti, et al. *Am J Prev Med. 1998*

What can we do?

We can ***prevent*** or ***reduce*** the health consequences of ACEs. Knowing the ***number*** of ACEs, without other details, helps us make recommendations for treatment.

We know that these questions are personal and sensitive. The first step is to get feedback from our CF families to understand the best way to ask about these ACEs.
